# Supplementary figures and images for: Conserved properties of Drosophila Insomniac link sleep regulation and synaptic function
Source: PLoS Genet. 2017 May 30;13(5):e1006815. doi: 10.1371/journal.pgen.1006815 (PMC5469494; doi:10.1371/journal.pgen.1006815)

**A**

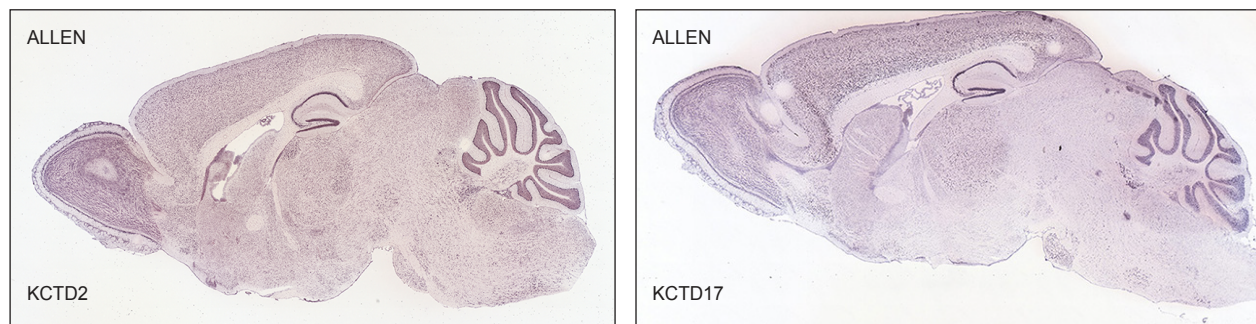

**B**

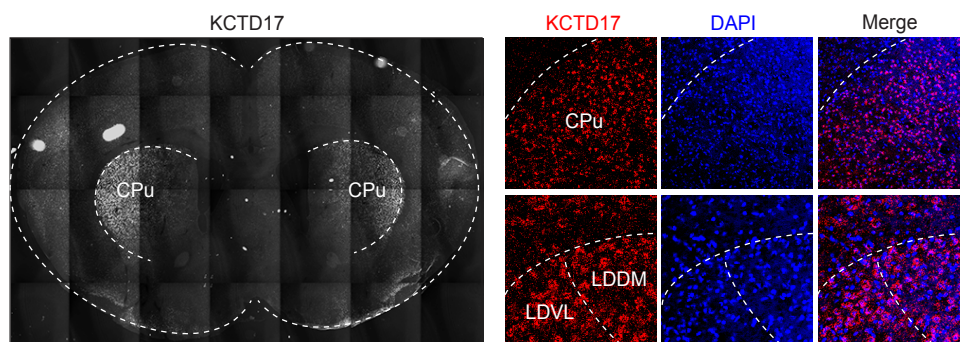

Figure S1

Supplement: S1 Fig — (A) Allen Brain Atlas in situ hybridization images for KCTD2 and KCTD17. Brain regions with KCTD2 signal include cortex, hippocampus, striatum, thalamus, hypothalamus, cerebellum, pons, and medulla. KCTD17 signal is more sparse and is present in cortex, hippocampus, striatum, thalamus, and cerebellum. KCTD17 probe in these experiments is complementary to predicted KCTD17 transcript isoforms v1, v2, and v5 (see S2 Fig and Materials and Methods). (B) In situ hybridization of mouse coronal brain section using a probe complementary to KCTD17 transcript isoforms 1, 2, 3, 4, and predicted variants v2 and v4. Signal is prominent in the caudate putamen (CPu) and thalamus, as shown for the laterodorsal thalamic nucleus (LDDM) and laterodorsal ventrolateral thalamus (LDVL), but weak or absent from cortex (left panel), suggesting that cortical KCTD17 signal in (A) may reflect differential expression of a nonoverlapping subset of KCTD17 transcript isoforms. (PDF) [file pgen.1006815.s001.pdf]

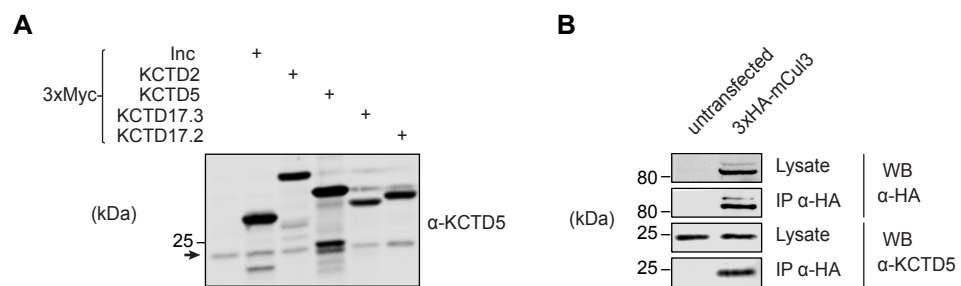

Figure S3

Supplement: S3 Fig — (A) Western blot of extracts from 293T cells transfected with indicated expression vectors and probed with anti-KCTD5. The first lane contains a control sample from cells transfected with empty vector and treated in parallel. Arrow indicates endogenous species corresponding to KCTD 2/5/17. (B) 293T cells transfected and immunoprecipitated as indicated. (PDF) [file pgen.1006815.s003.pdf]

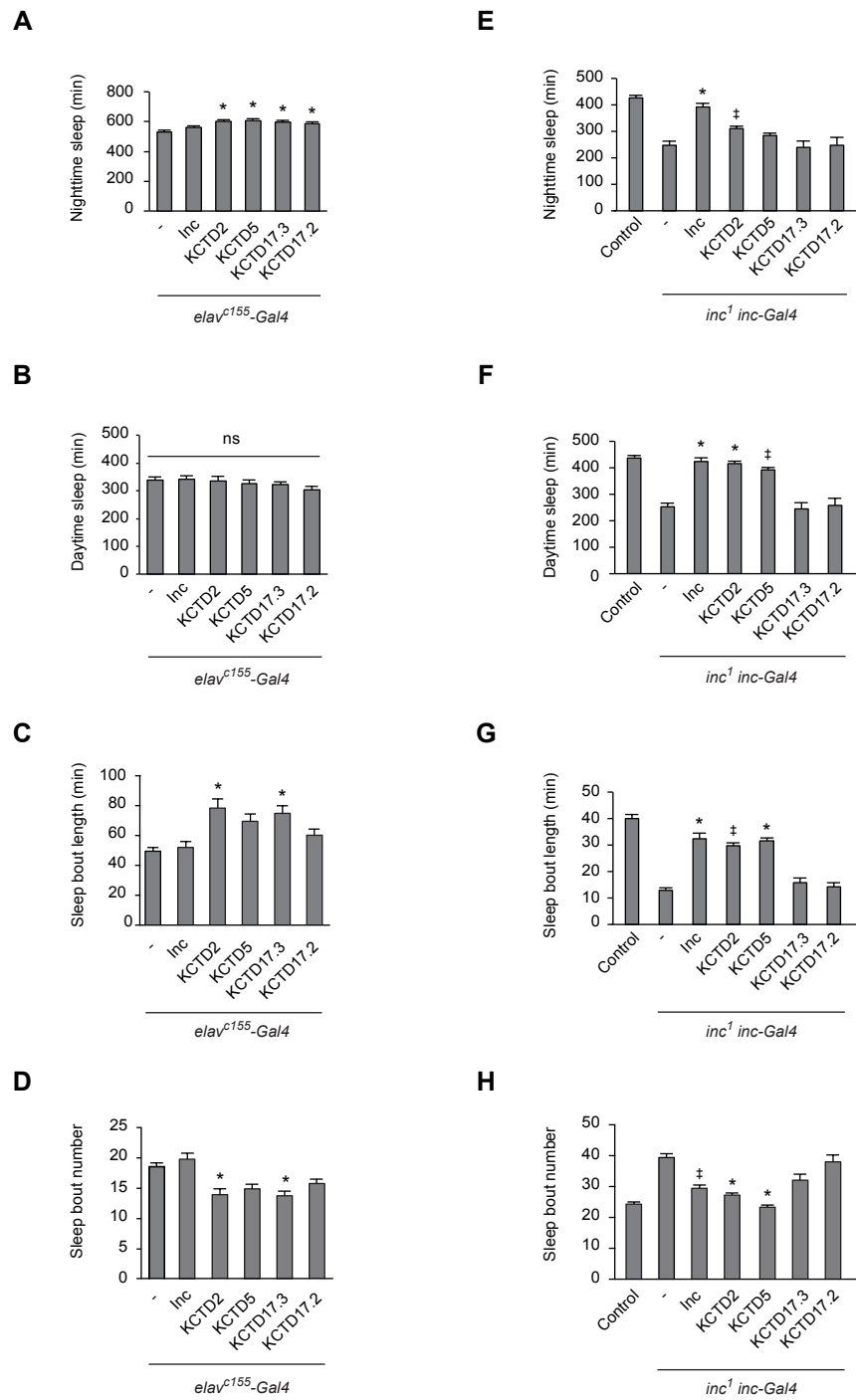

Figure S4

Supplement: S4 Fig — (A-D) Sleep parameters for animals expressing Inc and Inc orthologs panneuronally under elav-Gal4 control. n = 37–40 as in Fig 4C; * p < 0.01 compared to elav-Gal4 control; ns, not significant (p > 0.05). (E-H) Sleep parameters for inc1 inc-Gal4 animals expressing Inc and Inc orthologs. n = 18–157 as in Fig 4D. * p < 0.01 compared to inc1 inc-Gal4 animals, but not significantly different from wild-type control. ‡ p < 0.01 for comparisons to inc1 inc-Gal4 animals and to wild-type controls. For all panels, mean ± SEM is shown. (A and E) Nighttime sleep. (B and F) Daytime sleep. (C and G) Sleep bout length. (D and H) Sleep bout number. For all panels, animals are heterozygous for UAS transgenes. (PDF) [file pgen.1006815.s004.pdf]

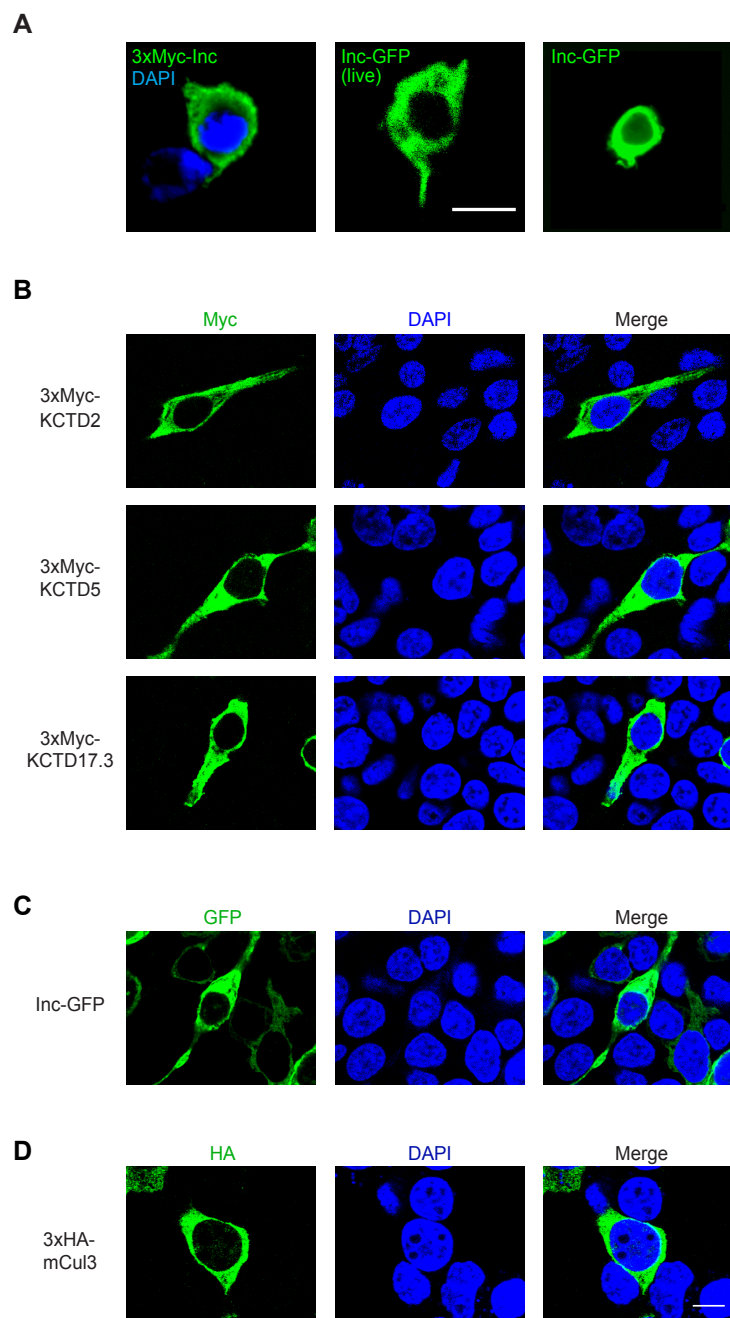

Figure S5

Supplement: S5 Fig — (A) Confocal micrographs of fixed S2 cell expressing 3×Myc-Inc (left panel), live S2 cell expressing Inc-GFP (middle panel), and fixed S2 cell expressing Inc-GFP (right panel). (B-D) Confocal micrographs of fixed 293T cells expressing indicated proteins bearing a 3×Myc tag (B), GFP tag (C), or 3×HA tag (D). Scale bar is 10 μM. (PDF) [file pgen.1006815.s005.pdf]

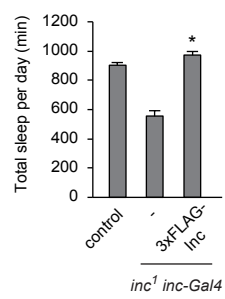

Figure S6

Supplement: S6 Fig — 3×FLAG-Inc rescues sleep duration of inc mutants when expressed under inc-Gal4 control. Animals are heterozygous for UAS-3×FLAG-Inc. n = 27–30, mean ± SEM is shown. * indicates not significantly different from wild-type animals and significantly different (p < 0.01) from inc1 inc-Gal4 control. (PDF) [file pgen.1006815.s006.pdf]

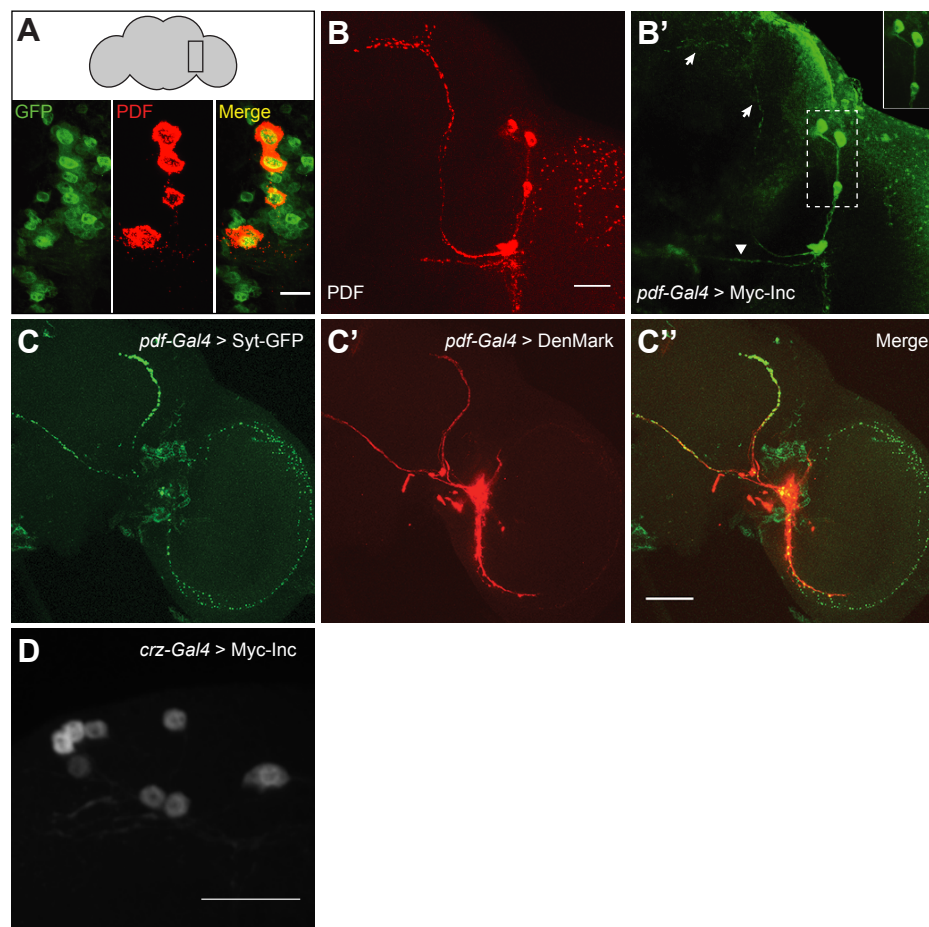

Figure S7

Supplement: S7 Fig — (A) inc-Gal4; UAS-nls-GFP / + adult brain stained with anti-GFP and anti-PDF antibodies. (B-B’) pdf-Gal4; UAS-Myc-Inc / + brain stained with anti-PDF (B) and anti-Myc (B’) antibodies. Arrows and arrowhead indicate Myc-Inc signal in dorsal and contralateral projections, respectively. In (B’), inset at lower gain shows primarily extranuclear Myc-Inc signal within PDF+ neuron cell bodies. (C-C”) pdf-Gal4; UAS-DenMark UAS-Syt-eGFP / + brain stained with anti-GFP and anti-dsRed antibodies. (D) Magnified Z-projection of crz-Gal4 / +; UAS-Myc-Inc / + left brain hemisphere from Fig 6C is shown at lower gain. anti-Myc signal in CRZ+ neuron cell bodies is primarily extranuclear or perinuclear. Scale bars represent 10 μm in (A), 25 μm in (B), 50 μm in (C), and 25 μm in (D). (PDF) [file pgen.1006815.s007.pdf]

**A**

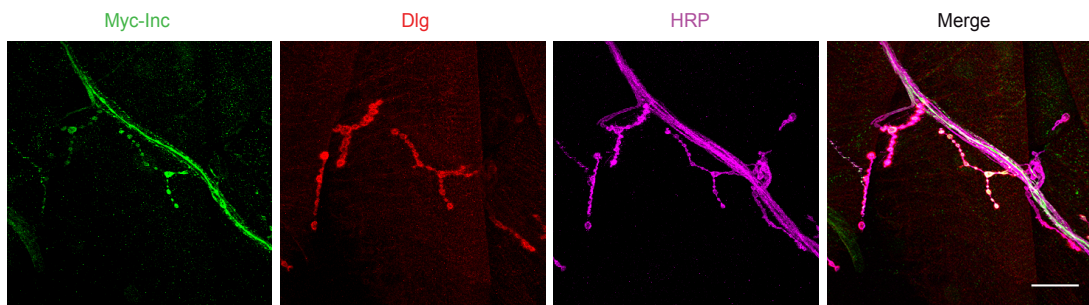

**B**

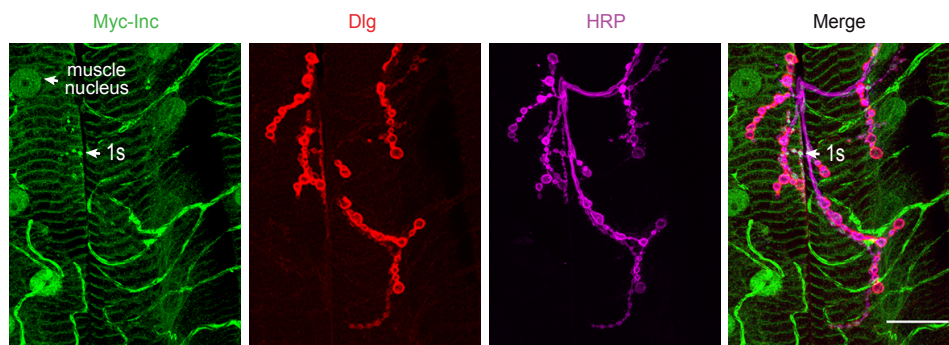

Figure S8

Supplement: S8 Fig — (A) Confocal micrograph of NMJ 4 from a female third instar inc-Gal4 / +; UAS-Myc-Inc / + larva. (B) Confocal micrograph of NMJ 6/7 from a male third instar inc-Gal4; UAS-Myc-Inc / + larva. Note higher level of Inc signal in muscle and HRP-negative trachea relative to larva shown in Fig 6F; this higher level of expression may reflect dosage compensation of the X-linked inc-Gal4 transgene in hemizygous males versus heterozygous females, or sex-specific position effects of the X-linked inc-Gal4 transgene insertion site. (PDF) [file pgen.1006815.s008.pdf]
