# Supplementary material for: Conserved properties of Drosophila Insomniac link sleep regulation and synaptic function
Source: PLoS Genet. 2017 May 30;13(5):e1006815. doi: 10.1371/journal.pgen.1006815 (PMC5469494; doi:10.1371/journal.pgen.1006815)
Supplement: S2 Fig — (A) Alignment of Inc and its mouse orthologs. Conserved BTB and C-terminal domains are indicated. Identical and similar residues are shaded in black and gray, respectively. (B) Analysis of alternatively spliced forms of mouse KCTD17 (mKCTD17). mKCTD2, mKCTD5, and mKCTD17 isoforms encoded by brain cDNAs are shown at top; predicted mKCTD17 splice variants from GenBank that may be conserved in vertebrates are shown beneath (v1, v4, v8). Other predicted variants are not depicted (v2, v3, v5, v6, v7, see Methods). Expression of at least some of these predicted variants in brain is suggested by in situ hybridization (S1A Fig) but requires further characterization. Alternative splicing yields variant C-termini, with color indicating residues shared by different isoforms. The sequence of a human KCTD17 isoform (hKCTD17.2) characterized in Kasahara et al [24] is shown at bottom. Deletion of underlined hKCTD17.2 residues abolishes interaction with trichoplein. Note that residues implicated in binding trichoplein are absent in mKCTD2, mKCTD5, mKCTD17.2, mKCTD17.3, and Inc. (PDF) [file pgen.1006815.s002.pdf]

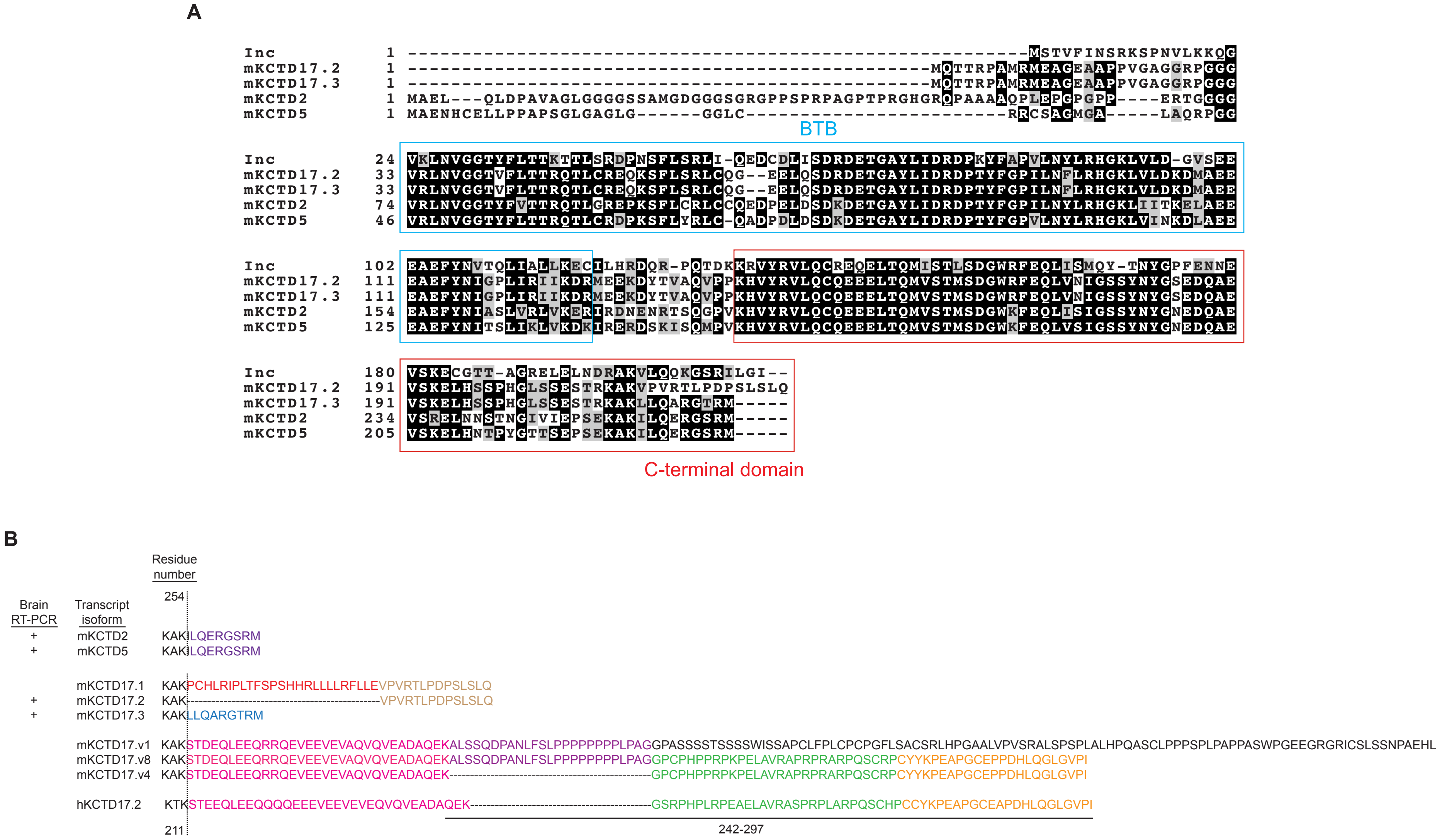
A
Inc 1 --MSTVFINSRKSPNVLKKQG
mKCTD17.2 1 --MQTTRPAMRMEAGEAAPPVGAGGRPGGG
mKCTD17.3 1 --MQTTRPAMRMEAGEAAPPVGAGGRPGGG
mKCTD2 1 MAEL---QLDPAVAGLGGGGSSAMGDGGGSGRGPPSPRPAGPTPRGHGRQPAAAAQPLEPGPGPP----ERTGGGG
mKCTD5 1 MAENHCELLPPAPSGLGAGLG------GGLC------------------------RRCSAGMGA-----LAQRPGG
BTB
Inc 24 VKLNVGGTYFLTTKTTLSRDPNSFLSRLI-QEDCDLISDRDETGAYLIDRDPKYFAPVLNYLRHGKLVLD-GVSEE
mKCTD17.2 33 VRLNVGGTVFLTTRQTLCREQKSFLSRLCQG--EELQSDRDETGAYLIDRDPTYFGPILNFLRHGKLVLDKDMAEE
mKCTD17.3 33 VRLNVGGTVFLTTRQTLCREQKSFLSRLCQG--EELQSDRDETGAYLIDRDPTYFGPILNFLRHGKLVLDKDMAEE
mKCTD2 74 VRLNVGGTYFVTTRQTLGREPKSFLCRLCCQEDPELDSDKDETGAYLIDRDPTYFGPILNYLRHGKLIITKELAEE
mKCTD5 46 VRLNVGGTYFLTTRQTLCRDPKSFLYRLC-QADPDLDSDKDETGAYLIDRDPTYFGPVLNYLRHGKLVINKDLAEE
Inc 102 EAEFYNVTQLIALLKECILHRDQR-PQTDKKRVYRVLQCREQELTQMISTLSDGWRFEQLISMQY-TNYGPFENNE
mKCTD17.2 111 EAEFYNIGPLIRIIKDRMEEKDYTVAQVPPKHVYRVLQCQEEELTQMVSTMSDGWRFEQLVNIGSSYNYGSEDQAE
mKCTD17.3 111 EAEFYNIGPLIRIIKDRMEEKDYTVAQVPPKHVYRVLQCQEEELTQMVSTMSDGWRFEQLVNIGSSYNYGSEDQAE
mKCTD2 154 EAEFYNIASLVRLVKERIRDNENRTSQGPVKHVYRVLQCQEEELTQMVSTMSDGWKFEQLISIGSSYNYGNEDQAE
mKCTD5 125 EAEFYNITSLIKLVKDKIRERDSKISQMPVKHVYRVLQCQEEELTQMVSTMSDGWKFEQLVSIGSSYNYGNEDQAE
Inc 180 VSKECGTT-AGRELELNDRAKVLQQKGSRILGI--
mKCTD17.2 191 VSKELHSSPHGLSSESTRKAKVPVRTLPDPSLSLQ
mKCTD17.3 191 VSKELHSSPHGLSSESTRKAKLLQARGTRM-----
mKCTD2 234 VSRELNNSTNGIVIEPSEKAKILQERGSRM-----
mKCTD5 205 VSKELHNTPYGTTSEPSEKAKILQERGSRM-----
C-terminal domain
B
Residue number
254
Brain RT-PCR
Transcript isoform
+ mKCTD2 KAKILQERGSRM
+ mKCTD5 KAKILQERGSRM
mKCTD17.1 KAKPCHLRIPLTFSPSHHRLLLLRFLLEVPVRTLPDPSLSLQ
+ mKCTD17.2 KAK--VPVRTLPDPSLSLQ
+ mKCTD17.3 KAKLLQARGTRM
mKCTD17.v1 KAKSTDEQLEEQRRQEVEEVEVAQVQVEADAQEKALSSQDPANLFSLPPPPPPPPLPAGGPASSSSTSSSSWISSAPCLFPLCPCPGFLSACSRLHPGAALVPVSRALSPSPLALHPQASCLPPPSPLPAPPASWPGEEGRGRICSLSSNPAEHL
mKCTD17.v8 KAKSTDEQLEEQRRQEVEEVEVAQVQVEADAQEKALSSQDPANLFSLPPPPPPPPLPAGGPCPHPPRPKPELAVRAPRPRARPQSCRPCYYKPEAPGCEPPDHLQGLGVPI
mKCTD17.v4 KAKSTDEQLEEQRRQEVEEVEVAQVQVEADAQEK--GPCPHPPRPKPELAVRAPRPRARPQSCRPCYYKPEAPGCEPPDHLQGLGVPI
hKCTD17.2 KTKSTEEQLEEQQQQEEEVEEVEVEQVQVEADAQEK--GSRPHPLRPEAELAVRASPRPLARPQSCHPCCYKPEAPGCEAPDHLQGLGVPI
211
242-297

Figure S2
